# Persistance du 5^ème^ arc aortique associé à une interruption de l’arche aortique

**DOI:** 10.11604/pamj.2017.27.265.4249

**Published:** 2017-08-09

**Authors:** Mahdi Ait Houssa, Noureddine Atmani, Mehdi Bamous, Abdessamad Abdou, Fouad Nya, Anis Seghrouchni, Brahim Amahzoune, Youssef El Bekkali, Mohamed Drissi, Abdelatif Boulahya

**Affiliations:** 1 Service de Chirurgie Cardiovasculaire, Hôpital Militaire Mohamed V, Rabat, Maroc; 2 Service de Réanimation, Hôpital Militaire Mohamed V, Rabat, Maroc

**Keywords:** Persistance du 5ème arc aortique, interruption de l´arche aortique, anomalies des arcs aortiques, chirurgie cardiaque, Persistence of the 5^th^ aortic arch, interruption of the aortic arch, abnormalities of the aortic arches, cardiac surgery

## Abstract

Les auteurs rapportent un cas de persistance du 5^ème^ arc aortique associé à une interruption totale de l'arche aortique. Ce cas clinique montre le piège diagnostic posé par la persistance du 5^ème^ arc aortique et son effet bénéfique hémodynamique. Le tableau clinique était trompeur en préopératoire en raison de la persistance des pouls fémoraux et des signes cliniques d'un shunt gauche-droite via un large canal artériel. Le diagnostic a été redressé en peropératoire grâce au monitorage de la pression artérielle par un cathéter placé dans l'artère fémorale.

## Abstract (en)

We report a case of persistence of the 5th aortic arch associated with total interruption of the aortic arch. This clinical case shows the diagnostic pitfall of the persistence of the 5th aortic arch and its beneficial hemodynamic effect. Preoperative clinical picture was misleading, due to the persistence of femoral pulses and clinical signs of left-to-right shunt via a wide ductus arteriosus. The diagnosis was intraoperatively adjusted on the basis of blood pressure monitoring using catheter placed into the femoral artery.

## Introduction

La persistance du 5^ème^ arc aortique est une anomalie congénitale extrêmement rare. La majorité des cas rapportés dans la littérature étaient diagnostiqués fortuitement et étaient associés à des malformations cardiovasculaires diverses [1]. La persistance du 5^ème^ arc aortique se présente sous forme d'un canal artériel qui nait de la portion distale de l'aorte thoracique ascendante et rejoint l'aorte thoracique descendante donnant l'aspect d'un dédoublement de la crosse aortique. Le siège de sa connexion distale définit une variété de présentation clinique selon le type de shunt établi; systémico-systémique ou systémico-pulmonaire. Nous rapportons un cas de persistance du 5^ème^ arc aortique associé à une interruption complète de l'arche aortique diagnostiquée en peropératoire et traité avec succès.

## Patient et observation

**Figure 1:**
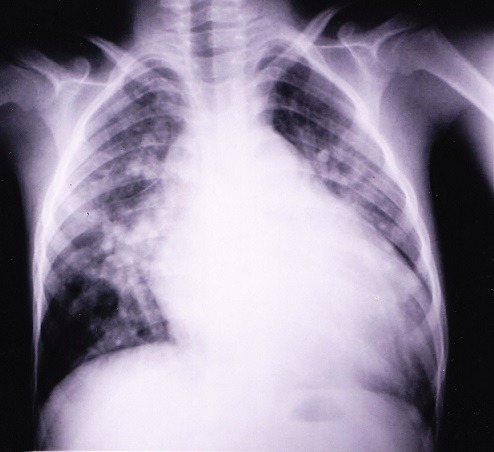
Radiographie pulmonaire de face montrant l’accentuation de la vascularisation pulmonaire

**Figure 2:**
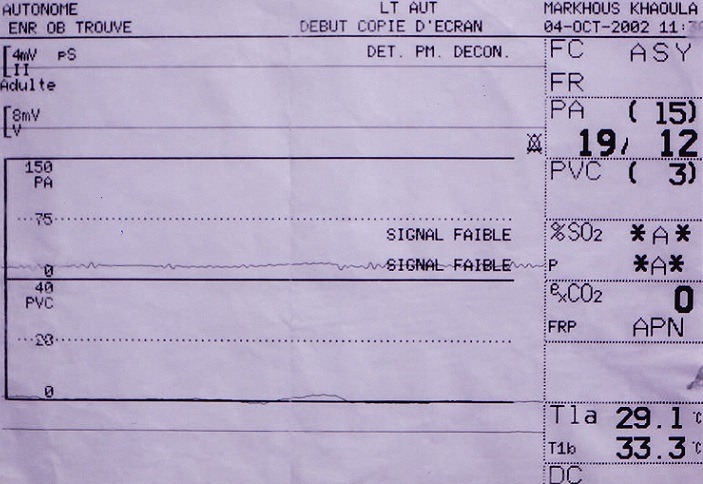
Amortissement de la courbe de pression après ligature du « CAP »

Une jeune fille de 6 ans avec un poids de 16 Kg a été admise dans le service de chirurgie cardiovasculaire de l'hôpital militaire d'instruction Mohammed V de Rabat pour prise en charge d'un rétrécissement aortique (RA) sous valvulaire associé à un canal artériel persistant (CAP). Ses antécédents ont été marqués par une hématémèse secondaire à une gastrite documentée par la fibroscopie et l'étude histo-pathologique. Elle rapportait des infections pulmonaires à répétition associés à une dyspnée d'effort. L'examen physique avait trouvé une pression artérielle à 90/40 mmHg, un pouls à 100 battements/mn. Tous les pouls périphériques étaient présents. L'auscultation avait objectivé un souffle systolique de rétrécissement aortique chiffré 3/6 associé à un souffle continu sous claviculaire gauche. L'ECG montrait un rythme régulier sinusal et une hypertrophie ventriculaire gauche. La radiographie thoracique a montré une cardiomégalie avec un rapport cardio-thoracique à 0,60 et une vascularisation pulmonaire accentuée (Figure 1). L'échocardiographie transthoracique a mis en évidence une dilatation des cavités cardiaques gauche et de l'artère pulmonaire (AP), un diaphragme fibreux sous valvulaire avec dilatation de l'aorte ascendante. Le gradient ventricule gauche (VG) - aorte maximal était à 55 mmHg. La pression artérielle pulmonaire systolique (PAPS) était de 60 mmHg. Il existait un shunt gauche-droite exclusif via un large canal artériel entre la crosse aortique et la bifurcation de l'AP. L'indication chirurgicale retenue était la ligature du CAP associé à une résection du diaphragme sous aortique. L'intervention chirurgicale a été réalisée sous anesthésie générale et par une stérnotomie médiane. Le monitorage de la pression artérielle a été fait par une artère sanglante fémorale à cause des difficultés de cathétériser les artères des membres supérieurs. La circulation extracorporelle (CEC) a été installée entre l'aorte ascendante et les veines caves. Avant le démarrage de la CEC, un gros canal a été disséqué et contrôlé, faisait communiquer la crosse aortique à la bifurcation de l'AP et ce gros vaisseau a été pris par large CAP. Après la mise en route de la CEC, la ligature du gros canal avait entrainé un amortissement manifeste de la courbe de PA fémorale (Figure 2) associé à une hyperpression au niveau de l'aorte ascendante. A ce moment, une dissection large de la bifurcation artérielle pulmonaire et de la région isthmique a été faite ce qui a permis de découvrir le vrai CAP qui est collé à l'AP gauche et se prolonge en bas par l'aorte descendante alors que la crosse aortique n'amorçait aucune courbure vers le bas et se prolongeait par l'artère sous clavière gauche après avoir donné le canal aberrant.

**Figure 3:**
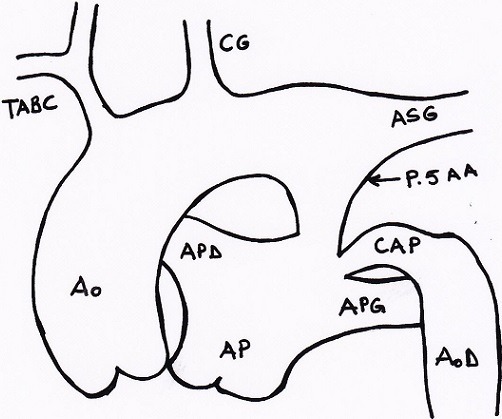
Schéma représentant le type A de la classification de Patton et Celoria

**Figure 4:**
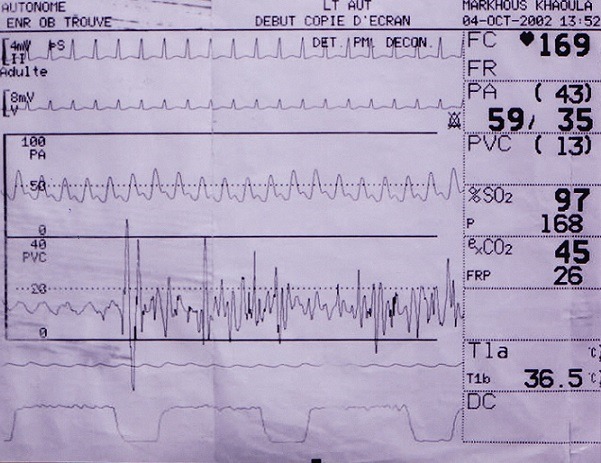
Reprise d’une courbe normale de la pression artérielle fémorale à la fin du montage

Ces anomalies étaient compatibles avec une interruption complète de l'arche aortique type A selon la classification de Patton et Celoria (Figure 3). Le vrai CAP a été alors ligaturé et sectionné et l'aorte thoracique descendante a été clampée le plus distale possible pour éviter toute tension sur l'anastomose. Le rétablissement de la continuité aortique a été réalisé par l'anastomose en termino-latéral de l'aorte descendante à la face inférieure de la crosse près de l'origine de la sous clavière gauche par un surjet continu au fil monobrin 5/0. Après remise en charge de l'anastomose, la courbe de la PA fémorale avait repris son amplitude normale (Figure 4). Durant la reconstruction de l'arche aortique, la perfusion coronaire et cérébrale ont été maintenues par un faible débit et la température rectale a été abaissée à 32°C. Après le rétablissement de la continuité aortique, la résection du diaphragme sous aortique a été faite sous clampage aortique et cardioplégie froide. Les durées de la CEC et du clampage aortique étaient respectivement 106 et 26 minutes. La sortie de la CEC avait nécessité de faibles doses de dobutamine (10 gamma/Kg/min) et le monoxyde d'azote (NO). La patiente a été extubée à la 7^ème^ heure postopératoire et elle a quitté l'hôpital au 10^e^ jour. Elle a été suivie régulièrement durant 42 mois et n'a présenté aucune complication (avec une évolution favorable). Le dernier contrôle avait trouvé un gradient de pression systolique entre les membres supérieurs et les membres inférieurs de 15 mmHg.

## Discussion

La persistance du 5^ème^ arc aortique est une malformation congénitale extrêmement rare. En raison des difficultés diagnostic, l'incidence exacte de cette anomalie reste inconnue. Gerlis dans une étude autopsique l'estime à 1cas/330 autopsie. [2]. Il existe une large controverse concernant l'existence du 5^ème^ arc aortique chez l'espèce humaine. En 1913, Brown [3] avait décrit l'existence du 5^ème^ arc aortique d'après l'étude embryologique chez le chat, en 1922, Buell [4] avait appuyé les données rapportées par Brown en décrivant la même constatation anatomique chez l'embryon de poulet. Le premier cas décrit embryologiquement dans l'espèce humaine a été fait par Huntington en 1919 [5], le premier cas clinique a été rapporté par Van Praagh en 1969 [6] en post mortem alors que le premier cas vivant a été décrit par T. Izukawa [7] en 1973 par aortographie. Dans l'embryologie humaine, l'aorte ventrale et l'aorte dorsale sont liés par des segments (canaux artériels) doublés (droite et gauche) appelés arcs aortiques. Certain arcs continuent leur développement alors que d'autres régressent puis disparaissent. La crosse de l'aorte et ses branches collatérales (TSA) naissent par fusion de la 3^ème^ et 4^ème^ paire d'arc aortique et de l'involution puis disparition du segment droit de l'aorte dorsale. La 6^ème^ paire d'arc aortique donne le tronc de l'artère pulmonaire, ses branches et le canal artériel persistant. Dans l'espèce humaine la 5^ème^ paire d'arc aortique régresse et disparaît précocement [8]. Toute anomalie de développement embryologique de ces arcs aortiques serait à l' origine d'une série d'anomalies congénitales de l'aorte et de ses branches principales. La persistance du 5^ème^ arc aortique donne un gros canal artériel anormal reliant la partie distale de la crosse aortique soit à l'aorte descendante soit à l'artère pulmonaire [9]. D'après Van Praagh, il existe 3 types de 5^ème^ arc aortique persistant [6]. Récemment, Oppido et Davies [10] avaient proposé une classification de la persistance du 5^ème^ arc aortique en 2 types: Le type A ou le gros canal persistant est responsable d'un shunt systémico-systémique, et le type B à l'origine d'un shunt systémico-pulmonaire. Le type A peut être subdivisé à trois sous groupes: A1: double arc aortique avec ou sans coarctation. A2: associé à une interruption de l'arche aortique. A3: avec une artère sous clavière gauche naissant de l'aorte ascendante ou du tronc artériel brachéo-céphalique. De même, le type B est subdivisé en trois sous groupes: B1 associé à une sténose pulmonaire ou atrésie pulmonaire, récemment, Gerard Holmes a publié le seul cas d'association à une tétralogie de Fallot [11]. B2: associé à une coarctation ou à une interruption de l'arche aortique. B3: associé à un hyper débit pulmonaire.

Cette classification a une implication clinique et thérapeutique. En effet, la persistance du 5^ème^ arc aortique peut être asymptomatique, restant ainsi méconnu et par conséquent il n y'a pas d'indication chirurgicale. C'est le cas du type A3 (avec une artère sous Clavière gauche naissant de l'aorte ascendante ou du tronc artériel brachéo-céphalique) comme le cas publié par Moes CA [12]. Ou le type A1 (double arc aortique sans obstacle de l'isthme; coarctation ou interruption) comme le cas publié par Gerlis [1]. Inversement, lorsque la persistance du 5^ème^ arc aortique est isolée ou associée à un hyper débit pulmonaire (type B3), la fermeture chirurgicale est justifiée. Dans les autres cas, la symptomatologie clinique et le traitement chirurgical dépendent des lésions cardiovasculaires associées. D'après l'étude des cas publiés, la persistance du 5^ème^ arc semble avoir un bénéfice hémodynamique. En effet, il joue le rôle d'une anastomose systémico-pulmonaire en cas de sténose de la voie pulmonaire comme le cas d'Oppido et Davies [10] et les cas de tétralogie de fallot publié par G. Holmès [11] et le cas de pentalogie de fallot rapporté par Furtado A.D [13]. En cas d'interruption totale de l'arche aortique, il assure une oxygénation adéquate de la partie inférieure du corps dont la seule issue du sang était le canal artériel persistant. Inversement, l'absence de l'obstacle sur la voie pulmonaire explique l'évolution rapide vers l'insuffisance cardiaque et l'hypertension artérielle pulmonaire particulièrement lorsqu'un autre shunt gauche droite lui est associé. Chez notre patiente il semble qu'un certain équilibre entre le shunt gauche droite via la persistance du 5^ème^ arc aortique et le shunt droite gauche via le CAP avait atténué la symptomatologie clinique et avait retardé le diagnostic. L'expression clinique est souvent précoce en raison des anomalies cardiovasculaires associées. dans des rares cas, le diagnostic a été fait tardivement comme le patient rapporté par Chaw-chi-CHIU [14] âgé de 7ans et le patient de Yong-Hong Zhao [15], agé de 9 ans. Ce dernier cas avait les mêmes lésions cardiovasculaires que notre patiente et le cas rapporté par Tehrai M [16]. L'interruption de l'arche aortique est rarement associée à la persistance du 5^ème^ arc aortique. Gerlis [2] dans une revue de littérature a trouvé seulement 2 cas d'interruption de l'arche aortique sur 21 cas de persistance du 5^ème^ arc aortique. A notre connaissance, depuis sa description par Van Praagh jusqu'à ce jour 29 cas de persistance du 5^ème^ arc aortique ont été rapportés dans la littérature en plus de notre observation. (Tableau 1) L'interruption de l'arche aortique est une malformation qui est souvent décrite dans le syndrome de Di-Georges due à une micro-délétion 22q11 [17]. Moor [18] avait soutenue l'hypothèse hémodynamique comme facteur favorisant le développement de l'interruption de l'arche aortique. Pour la persistance du 5^ème^ arc aortique, l'étiologie n'est pas encore élucidée en dehors du seul cas rapporté par Lawrence s'inscrivant dans le cadre d'un syndrome poly malformatif (dysmorphie facial, fente palatine, persistance du 5^ème^ arc aortique), dont la cause était une exposition à la trimethadione qui est connue par son action tératogène [19]. Le 5^ème^ arc aortique surtout dans le type B est souvent de gros calibre, et pris pour un large canal artériel. Le diagnostic est facile entre les mains d'un échocardiographiste expérimenté, mais il faut y penser dans des situations douteuses et compléter l'exploration par une imagerie par résonnance nucléaire (IRM) ou une angiographie. L'originalité de notre observation est ce piège diagnostique posé par la persistance du 5^ème^ arc aortique pris comme un gros canal artériel persistant et la méconnaissance de l'interruption de l'arc aortique en raison de la présence des pouls fémoraux et c'est le monitorage de la pression artérielle par un cathéter fémoral qui a permis de redresser le diagnostic.

**Tableau 1:** Les différents cas de persistance du 5^ème^ arc aortique publiés dans la littérature

Auteur	Nombre de cas	sexe	Age	Lésions CVx associées	Référence	Année
T. Izukawa	1	F	4,5mois	CAP		1973
T. Izukawa	1	M	1jour	Bicuspidie aortique, artere coronaire unique naissant du sinus droit, coarctation aortique		1973
F.J. Macartney	+		4 ans	APSO		1974
A.Cabrera	1	M	1jour	CAP		1985
Antonio. J. Marinho	1	M	7mois	T4F		1998
Jeih-Neng-Wang	1	F	1jour	VDDI, CAP, AP gauche naissant de l’aorte ascendante		1999
Chaw-Chi-Chiu	1	M	4 ans	-		2000
Chaw-Chi-Chiu	1	F	1jour	CIV large		2000
Cheong Lim	1	F	2mois	Truncus arteriosus, interruption de l’arche type B, CIV		2002
Mao Sheng Hwang	1	M	2mois	-		2003
SG. Yang	+		3mois	FOP, CAP		2003
M.L. Lee	+		5jours	T4F, atrésie pulmonaire, Sd Di George		2003
Y. Isomatsu	1	F	28ans	CoAo		2004
Daniel.J. Bibardino	1	F	11mois	-		2004
HJ. Park	+		3ans	Persistance du 5^ème^ AA dédoublé (1ér cas decrit)		2005
ML. Lee	4					2006
J. Iwase	1		2jours	CAP, CoAo		2006
MK. Krishnamoorthy	1			T4F		2006
SJ. Carroll	1		9jours	CAP, CoAo		2006
Sufara Khan	1	F	3jours	CoAo (arche dédoublé=5^ème^ AA)		2006
Sufara Khan	1	M	1mois	Sténose de l’AP gauche, Atrésie alimentée par 5^ème^ AA		2006
Sufara Khan	1	M	2jours	Truncus arteriosus, CIV, CIA, VCSG dans le SC		2006
MH. Lee	1		1jour	TGV, Atrésie pulmonaire		2007
J. Kirsh	1		7ans	Arche aortique double		2007
Y. Zong	1		9ans	Interruption de l’AA type A		2007
Y. Zong	1		3ans	Interruption de l’AA type B		2007
Y. Zong	1		11mois	Interruption de l’AA type A		2007
Y. Zong	1		6mois	Interruption de l’AA type A		2007
J. Bernhermer	1	F	1jour	Coronaire unique naissant du sinus droit, VDDI, Atrésie pulmonaire		2007
J. Bernhermer	1	M	17mois	Interruption de l’AA type A		2007
A. Koch	1	F	5sem	Sténose del’AP gauche		2007
Yong-Hong-Zhao	1	M	9ans	Interruption de l’AA type A		2007
Yong-Hong-Zhao	1	M	7mois	Interruption de l’AA type A		2007
G. Holmes	1	F	7sem	T4F		2007
Giuseppa Sautoro	1	M	2jours	CoAo, CAP		2009
Kligerman. S						2009
Raghavan. Subramanyan	1	F	1an	CIV		2010
Chang-Hsien.Yu	1	M	2mois	CoAo		2010
RR. Linhares	+		47ans	-		2011
AD. Furtado	1	M	21mois	T4F, CIA		2011
Zahra Khajali	1	M	20ans	CoAo		2011
Mohamad Tehrai	1	F	64ans	-		2012
G. Holmes	1	F	7sem	T4F		2012
P. Zartner	1	F	1jour	CAP		?

Légende : **AA** arc aortique, **M** : male, **F** : femelle, **APSO** : atrésie pulmonaire à septum ouvert, **T4F** : tétralogie de Fallot, **VDDI** : ventricule droit à double issue, **FOP** : foramen ovale perméable, **CoAo** : coarctation de l’aorte, **VCSG** : veine cave supérieure gauche, **SC** : sinus coronaire, **TGV** : transposition de gros vaisseaux

## Conclusion

La persistance du 5^ème^ arc aortique est une anomalie congénitale peu fréquente et rarement isolée, le tableau clinique est très variable selon les anomalies associés mais le diagnostic est facile entre les mains d'un échocardiographiste expérimenté.

## Conflits d’intérêts

Les auteurs ne déclarent conflit d’intérêts.
